# Measurable residual disease in chronic lymphocytic leukemia: expert review and consensus recommendations

**DOI:** 10.1038/s41375-021-01241-1

**Published:** 2021-06-24

**Authors:** William G. Wierda, Andrew Rawstron, Florence Cymbalista, Xavier Badoux, Davide Rossi, Jennifer R. Brown, Alexander Egle, Virginia Abello, Eduardo Cervera Ceballos, Yair Herishanu, Stephen P. Mulligan, Carsten U. Niemann, Colin P. Diong, Teoman Soysal, Ritsuro Suzuki, Hoa T. T. Tran, Shang-Ju Wu, Carolyn Owen, Stephan Stilgenbauer, Paolo Ghia, Peter Hillmen

**Affiliations:** 1 MD Anderson Cancer Center, Houston, TX USA; 2 Haematological Malignancy Diagnostic Service, Leeds, UK; 3 Hôpital Avicenne, AP-HP, UMR Université Paris13/INSERM U978, Bobigny, France; 4 The St. George Hospital, Sydney, NSW Australia; 5 Hematology, Oncology Institute of Southern Switzerland, Bellinzona, Switzerland; 6 Dana-Farber Cancer Institute and Harvard Medical School, Boston, MA USA; 7 Department of Internal Medicine III with Haematology, Medical Oncology, Hemostaseology, Infectiology and Rheumatology, Oncologic Center, Paracelsus Medical University, Salzburg, Salzburg Cancer Research Institute - Laboratory for Immunological and Molecular Cancer Research (SCRI-LIMCR), Cancer Cluster Salzburg, Salzburg, Austria; 8 Hospital de San José, Bogota, Colombia; 9 Instituto Nacional de Cancerologia/Médica Sur Fundación Clínica, Mexico City, Mexico; 10 Tel-Aviv Sourasky Medical Center and Sackler Medical School, Tel Aviv, Israel; 11 Royal North Shore Hospital, Sydney, NSW Australia; 12 Rigshospitalet, Copenhagen, Denmark; 13 Parkway Cancer Centre, Singapore, Singapore; 14 Cerrahpasa Faculty of Medicine, Istanbul University-Cerrahpasa, Istanbul, Turkey; 15 Shimane University Hospital, Shimane, Japan; 16 Akerhus University Hospital, Lørenskog, Norway; 17 National Taiwan University Hospital, Taipei, Taiwan; 18 Tom Baker Cancer Centre, Calgary, AB Canada; 19 Internal Medicine III, Ulm University, Ulm and Internal Medicine 1, Saarland University, Homburg, Germany; 20 Università Vita-Salute San Raffaele and IRCCS Ospedale San Raffaele, Milan, Italy; 21 Leeds Teaching Hospitals, NHS Trust, Leeds, UK

**Keywords:** Chronic lymphocytic leukaemia, Medical research

## Abstract

Assessment of measurable residual disease (often referred to as “minimal residual disease”) has emerged as a highly sensitive indicator of disease burden during and at the end of treatment and has been correlated with time-to-event outcomes in chronic lymphocytic leukemia. Undetectable-measurable residual disease status at the end of treatment demonstrated independent prognostic significance in chronic lymphocytic leukemia, correlating with favorable progression-free and overall survival with chemoimmunotherapy. Given its utility in evaluating depth of response, determining measurable residual disease status is now a focus of outcomes in chronic lymphocytic leukemia clinical trials. Increased adoption of measurable residual disease assessment calls for standards for nomenclature and outcomes data reporting. In addition, many basic questions have not been systematically addressed. Here, we present the work of an international, multidisciplinary, 174-member panel convened to identify critical questions on key issues pertaining to measurable residual disease in chronic lymphocytic leukemia, review evaluable data, develop unified answers in conjunction with local expert input, and provide recommendations for future studies. Recommendations are presented regarding methodology for measurable residual disease determination, assay requirements and in which tissue to assess measurable residual disease, timing and frequency of assessment, use of measurable residual disease in clinical practice versus clinical trials, and the future usefulness of measurable residual disease assessment. Nomenclature is also proposed. Adoption of these recommendations will work toward standardizing data acquisition and interpretation in future studies with new treatments with the ultimate objective of improving outcomes and curing chronic lymphocytic leukemia.

## Introduction

In patients with chronic lymphocytic leukemia (CLL) treated with fixed-duration regimens, such as chemoimmunotherapy (CIT), end-of-treatment response by standard criteria correlates with time-to-event endpoints, including progression-free (PFS) and overall survival (OS) [1–5]. Complete remission (CR) by National Cancer Institute-sponsored Working Group/international Workshop on Chronic Lymphocytic Leukemia (NCI-WG/iwCLL) criteria is associated with superior outcomes. Achieving this depth of response has been the therapeutic goal. However, CR is not disease eradication. Advanced diagnostic methods have enabled detection of very low levels of disease in peripheral blood (PB) and bone marrow (BM). Low but measurable persistent CLL is present in many CIT-treated patients, including those achieving CR.

CLL cells are found in lymphoid tissues and circulate through blood and lymphatics. Measurable residual disease (MRD; often referred to as “minimal residual disease”) is distinct from standard response, providing additional independent prognostic information. MRD is a sensitive reflection of disease burden during and after fixed-duration treatment and has been correlated with PFS and OS (Table 1). Because Bruton tyrosine kinase inhibitor- and PI3K inhibitor-based treatments are continuous and responses are not deep, achieving undetectable-MRD (U-MRD) with such monotherapies is uncommon [6]. Depth of remission with BCL2 inhibitor-based monotherapy (venetoclax) or in combination with a CD20 monoclonal antibody (mAb) is greater, and such combination regimens are of fixed-duration. Furthermore, data with fixed-duration combined Bruton tyrosine kinase inhibitors and BCL2 inhibitors (with or without CD20 mAb) indicate high CR and U-MRD rates [7–12]. Further data are needed to clarify the association of MRD status with time-to-event endpoints in these regimens.

**Table 1:** Studies assessing the correlation between MRD status and time-to-event outcomes in patients with CLL.

Study	Line	Type	*N*	Agent	Assay	Tissue	Outcome	Result
Egle et al. [72]	First	Ph 1/2	39	FR + len	Flow MRD3	PB	mPFS	76.1 vs 46.4 mo, *P* = 0.007
Abrisqueta et al. [18]	First	Ph 2	59	R-FCM	Flow MRD4	PB BM	4-yr PFS	PB: 89.5% vs 27% (*P* < 0.01) BM: 86% vs 60% (*P* = 0.027)
Appleby et al. [37]	First	Ph 2	52	FCR	Flow MRD4	BM	mTTF	85.3 vs 59.2 mo (*P* = 0.0008)
Fischer et al. [20]	First	Ph 2	45	BR	Flow MRD4	PB	mEFS	32.4 mo^a^ vs 11.8 mo^b^ vs NR^c,^ *P* < 0.001
							mOS	23.2 mo^a^ vs NR^b^ vs NR^c^
Frankfurt et al. [38]	First	Ph 2	30	AlemR	Flow MRD4	BM	mPFS	41.3 vs 16.9 mo (*P* = 0.026)
Kay et al. [3]	First	Ph 2	52	RC + pent	Flow MRD2	PB	PFS	HR 0.22, *P* = 0.003
Short et al. [73]	First	Ph 2	60	FCR3	Flow MRD2	PB	mTTP	No significant difference
Strati et al. [23]	First	Ph 2	161	FCR	Flow MRD4	BM	PFS	HR 0.1, *P* = 0.04
							OS	HR 0.7, *P* = 0.05
Thompson et al. [24]	First	Ph 2	300	FCR	PCR MRD4	PB/BM	mPFS	13.7 vs 4.0 yr
Böttcher et al. [19]	First	Ph 3	493	FCR vs CF	Flow MRD4	PB	PFS	HR 2.49, *P* < 0.0001
							OS	HR 1.36, *P* = 0.36
Goede et al. [44]	First	Ph 3	231	GClb arm	ASO-PCR	PB/BM	mPFS	NR vs 19.4 mo
Greil et al. [74]	First and second	Ph 3	263	CIT+/- R-maintenance	Flow MRD4	PB/BM	PFS	HR 0.4 by PB MRD4, *P* < 0.0001 HR 0.26 by BM MRD4, *P* < 0.0001
Kovacs et al. [21]	First	Pooled Ph 3	554	FCR vs CF FCR vs BR	Flow MRD4	PB	mPFS	60.7 vs 54.2 vs 35.4 vs 20.7 mo for U-MRD CR, U-MRD PR, MRD+ CR, and MRD+ PR
							mOS	NR vs NR vs NR vs 72.1 mo for U-MRD CR, U-MRD PR, MRD+ CR, and MRD+ PR
Santacruz et al. [22]	First	Retro	255	Any	Flow MRD4	PB/BM	mTFS	76 vs 16 mo, *P* < 0.001
							mOS	108 vs 78 mo, *P* = 0.014
Jones et al. [46]	R/R	Ph 2	57	Ven	Flow MRD4	PB	PFS	HR 0.23, *P* = 0.021
Moreton et al. [40]	R/R	Ph 2	33	Alem	Flow MRD4	BM	mTFS	NR vs 20 mo for U-MRD CR and MRD-positive CR, respectively, *P* < 0.0001
							mOS	NR vs 60 mo for U-MRD CR and MRD-positive CR, respectively, *P* = 0.0007
Fraser et al. [7]	R/R	Ph 3	578	Ibr + BR vs PBO + BR	Flow MRD4	PB/BM	36-mo PFS	Ibr + BR: 88.6% vs 60.1% PBO + BR: 54.5% vs 11.2%
Kater et al. [61]	R/R	Ph 3	276	VenR vs BR	Flow MRD4 ASO-PCR	PB	PFS	VenR: HR 0.48 (U-MRD vs b), HR 0.15 (U-MRD vs a) BR: HR 0.44 (U-MRD vs b), HR 0.08 (U-MRD vs a)
Stilgenbauer et al. [42]	Any	Ph 2	158	Ven	Flow MRD4	PB	18-mo PFS	78% vs 51%
Kwok et al. [39]	Any	Retro	133	Any	Flow MRD4	PB	mPFS	7.6 vs 3.3 vs 2.0 yr for U-MRD, b, and a, respectively
							mOS	10.6 vs 5.3 vs 3.6 yr for U-MRD, b, and a, respectively

^a^MRD ≥ 0.01.

^b^MRD ≥ 0.0001 and < 0.01.

^c^MRD < 0.0001.

*Alem* alemtuzumab, *AlemR* alemtuzumab and rituximab, *ASO-PCR* allele-specific oligonucleotide polymerase chain reaction, *BM* bone marrow, *BR* bendamustine and rituximab, *CF* cyclophosphamide and fludarabine, *CR* complete remission, *EFS* event-free survival, *Flow* flow cytometry, *FCM* fludarabine, cyclophosphamide, and mitoxantrone, *FCR* fludarabine, cyclophosphamide, and rituximab, *FR* fludarabine and rituximab, *G* obinutuzumab, *len* lenalidomide, *m* median, *MRD* measurable residual disease, *MRDx* MRD with an assay of 10^−x^ sensitivity, *NR* not reached, *OS* overall survival, *PB* peripheral blood, *pent* pentostatin, *PFS* progression-free survival, *Ph* phase, *RC* rituximab and cyclophosphamide, *Retro* retrospective analysis, *R-FCM* rituximab, fludarabine, cyclophosphamide, and mitoxantrone, *R/R* relapsed/refractory, *TFS* treatment-free survival, *TTF* time-to-treatment failure, *TTP* time to progression, *U-MRD* undetectable-measurable residual disease, *Ven* venetoclax, *VenR* venetoclax and rituximab.

Given its utility in evaluating depth of response and identifying treatment superiority in randomized trials, MRD determination is increasingly adopted in CLL trials, frequently as a co-primary or secondary endpoint [13]. Although PFS and OS remain regulatory endpoints, MRD status is an important surrogate approved by the European Medicines Agency as an intermediate endpoint for CLL trials. iwCLL guidelines recommend that “in clinical trials aimed at maximizing the depth of remission, the presence of MRD after therapy should be assessed [14].” It is, however, not yet applicable to standard community care.

As highly effective treatments achieving deep remission emerge, it becomes necessary to standardize new methods for assessing disease and response. Many relevant questions must be addressed—e.g., whether it is optimal to assess MRD in PB or BM, at which timepoint(s) to evaluate MRD, by which analysis method, and at what sensitivity. These issues have been touched on by the iwCLL and US and European regulatory agencies [14–16].

Here, we present a consensus document from an international, multidisciplinary, 174-member panel convened to assemble critical questions on key issues pertaining to MRD in CLL, review available data, develop unified answers with local expert input, and provide recommendations for future efforts.

## Methods

An international steering committee (ISC) was convened in Paris in March 2017. Key topics regarding the assessment and utility of MRD in CLL were identified, and a set of 84 pertinent questions was drafted. National Faculties (Supplementary Appendix) subsequently ranked these questions by importance (“How important does the National Faculty think this question is with regard to integrating MRD measurement into clinical practice?”) and timing (“How soon does the National Faculty predict that the answer to this question would have an impact on clinical practice?”). The sum of the scores for each question determined its ranking. An initial set of answers to the 13 highest-ranking questions based on ISC opinion and literature review was drafted and then refined by the National Faculties at a series of local/regional meetings (Supplementary Fig. S1). National Faculties voted on level of agreement with draft answers on a scale of 1–9, with agreement defined as ≥75% voting in the 7–9 range. If agreement was lacking after the first vote, the answer could be refined after discussion and National Faculties could vote a second time. If agreement was not achieved after a second vote, then no agreement was recorded. The consolidated feedback was reviewed by the ISC in Amsterdam in March 2018. Answers were finalized at a June 2018 meeting of the ISC and additional advisors (Supplementary Appendix) in Stockholm. The resulting report provided the basis for the consensus document presented herein. All authors reviewed/approved the submitted version of this manuscript.

The ISC was composed of 8 members (authors WGW, AR, FC, XB, JRB, PG, SS, PH), and the National Faculty was composed of 166 members for a total 174-member panel. ISC members were selected by AbbVie Global Medical Affairs after consideration of key markets, recognized international expertise, and any recommendations by another ISC member. National Faculties were nominated by AbbVie affiliates and reviewed by AbbVie Global Medical Affairs and the ISC. National Faculty selection criteria included the following: certified hematologist/oncologist; medical, clinical, and/or professional experience and scientists conducting relevant research; national, regional, or local distinction; credentials, including knowledge and experience in the applicable therapeutic or business area; and progressive approach to patient care.

## Nomenclature

Recommended standardized nomenclature for MRD is provided (Table 2). Although “MRD” is often defined as “minimal residual disease,” the term “minimal” is subjective. “Measurable residual disease,” which is unambiguous when the detection limit is specified, is recommended. The MRD report should either state the percentage of disease involvement in the specified tissue or that disease was not detectable. In either situation, it is critical that the report also provides the detection limit for the sample, because this will depend on both the assay used as well as the sample quality, primarily based on number of cells or amount of DNA available for analysis. A categorical measure indicating the upper limit of MRD should also be noted. We recommend “MRD4” to identify cases with <10^−4^ MRD (<1 CLL cell per 10 000 leukocytes, or <0.01%), “MRD5” to identify cases with <10^−5^ MRD (<1 CLL cell per 100,000 leukocytes, or <0.001%), etc.

**Table 2:** Recommended nomenclature for reporting measurable residual disease in CLL.

Recommended	Rationale
Measurable residual disease (MRD)	Replaces “minimal” residual disease as a more objective term
Undetectable-MRD (U-MRD)	As a general term, replaces MRD negative or MRD- as a more accurate term in cases where MRD threshold is not specified
MRD4, MRD5, etc.	Specifies upper limit of disease (e.g., MRD4 denotes <0.01%/<10^-4^ disease, MRD5 < 0.001%/<10^−5^ disease, etc) for an individual sample or for a group of patients in clinical trial reporting
Detectable (d) or undetectable (u) within an MRD category	Detectable = residual disease is below the stated threshold but measurable above the next MRD threshold. Undetectable = residual disease is not detectable, but the assay/sample is not suitable for detection of disease at the next threshold MRD4d: < 0.01%/10^−4^ but ≥0.001%/10^−5^ MRD4u: < 0.01%, assay limit of detection does not reach 0.001%/10^−5^
Always report assay method (e.g., Flow) and analysis technique (e.g., ERIC-FC)	Results may differ by assay method even for assays with identical sensitivity
Always report tissue assayed (e.g., PB, BM)	MRD may differ in different tissues from the same patient/timepoint
In clinical trials, always report MRD rate as percentage U-MRD in ITT population	Avoids confusion with the rate in the MRD-tested population, e.g., MRD4 rate = number of patients with <0.01% MRD as a percentage of the ITT population

*BM* bone marrow, *CLL* chronic lymphocytic leukemia, *Flow* flow cytometry, *ITT* intention-to-treat, *PB* peripheral blood.

“Undetectable-MRD” (U-MRD) is preferable to “MRD-” or “MRD negative” as a general term to describe the inability to detect measurable disease at a specified reporting threshold, because disease may be detectable below this level. Because it is now common to use an assay with better sensitivity than the reporting threshold (e.g., clinical trials reporting the MRD4 rate often use an assay with a detection limit of 1 CLL cell in 100,000 or 1 million leukocytes), it is more informative to follow the approach recommended by the CML community [17] and use detectable/undetectable to provide additional information on assay sensitivity and whether disease is detectable below the reporting threshold. So, the category “MRD4” would indicate MRD < 0.01%/<1 CLL cell per 10,000 leukocytes but does not specify whether disease is detectable or not below this level. MRD4d (detectable) indicates that an assay capable of detecting disease at the 0.001% threshold and residual disease is above this level but <0.01% (between 10^−4^ and 10^−5^). MRD4u (undetectable) indicates that residual disease is <0.01%, but the assay was not capable of detecting 0.001% disease due to assay or sample limitations.

MRD level may differ in the PB compared with that in the BM and so the sampled tissue (e.g., PB, BM) must be specified. The method used to determine MRD (e.g., flow cytometry [flow], polymerase chain reaction [PCR], next-generation sequencing [NGS]) should be specified. When reporting U-MRD rates for clinical trials, rates should be calculated based on the full intention-to-treat population. Missing values should be counted as positive by default; other ways of reporting must be clearly explained.

## Quantifying MRD

### MRD methodology

With improved methodology, the sensitivity at which CLL cells can be measured continues to increase. Limitations exist regarding sample volume, assay time, and use of cellular versus molecular assays. The minimum sensitivity limit for response assessment is 10^−4^, demonstrated to be an independent prognostic factor in patients treated with first-line CIT [18–24]. Assays require prospective technical validation and must undergo cross-laboratory standardization with external quality assurance (EQA) procedure [25, 26]. Available assays include ≥4 color flow cytometry and immunoglobulin heavy-chain variable region (IGHV) real-time quantitative PCR (RQ-PCR) capable of quantifying MRD at the 10^−5^ level (Table 3). Still more sensitive technologies undergoing validation include high-throughput sequencing/NGS and droplet digital PCR [27, 28].

**Table 3:** Methods of MRD detection.

Method	Sensitivity	Comment	References
4-color Flow	10^−4^	≥10^7^ fresh leukocytes needed; assay useful in CD20 regimens has been reported [75]	Rawstron [25]
≥6-color Flow	10^−5^	≥2 × 10^6^ fresh leukocytes needed	Rawstron [27, 28, 71]
8-color Flow	10^−6^	–	Letestu [76]
10-color Flow	10^−5^	–	Sartor [77]
ASO *IGH* RQ-PCR	10^−5^	Patient-specific primers needed	Böttcher [32]
ddPCR^TM^	10^−5^	Patient-specific primers needed; no CLL data reported	Drandi [27]
clonoSEQ^®^ Assay	10^−6^	Multiplex polymerase chain reaction and next-generation sequencing [78]	Thompson [79]

*ASO IGH RQ-PCR* allele-specific oligonucleotide immunoglobulin heavy locus polymerase chain reaction, *CLL* chronic lymphocytic leukemia, *ddPCR* droplet digital PCR, *Flow* flow cytometry.

In clinical trials, European Research Initiative in CLL (ERIC)-compliant flow and EuroMRD-compliant RQ-PCR are most common, typically performed at specialized centers [12, 25, 29–31]. For flow cytometry, the four-color assay is the historical gold standard, but six- and eight-color flow are also available. Four-color flow (CD5/CD19 with CD20/CD38, CD81/CD22, and CD79b/CD43) and RQ-PCR have undergone clinical validation and cross-laboratory standardization [25, 26, 32]. Flow cytometry is optimal for rapid turnaround, whereas RQ-PCR requires a pretreatment sample to determine the target sequence and patient-specific primers, making it more suitable for batch processing (e.g., at trial completion). However, samples <48 h old are preferred for flow cytometry, though can be accepted to <72 h [33], whereas PCR and NGS can use stored DNA.

#### Consensus recommendations

*Only validated assays are recommended. Validated methods include ERIC-compliant flow cytometry and EuroMRD-compliant RQ-PCR. The choice of assays depends upon the rationale for MRD determination. The minimum sensitivity required for regulatory approval is MRD4 (10*^*−4*^*), whereas evaluating curative approaches may require the most sensitive method available, based on local availability and/or economic restrictions. When reporting MRD data, the quantification and/or detection limit should be stated for each sample. Method validation and standardization information should be provided. Providing quantitative results for detectable MRD is also recommended*.

### MRD assay requirements

Standardization is the process of developing and implementing technical standards based on multiparty consensus. Documented standards for flow (ERIC) and RQ-PCR (EuroMRD) have been reported [25, 26]. Harmonization is the process of coordinating different systems, creating minimum requirements or standards to ensure global reproducibility [34]. Validation comprises analytical validation (for accuracy, precision, and reproducibility) and clinical validation, ensuring that the assay effectively associates with clinical outcome [34]. Verification evaluates whether a validated assay meets the required standard. Quality assurance (QA) or conformity schemes are required for localized testing [15].

#### Consensus recommendations

*Harmonized MRD testing is essential for globally reproducible results. MRD testing may be performed in central laboratories, regional centers, or local hospital laboratories, provided the assay meets accepted standards. When using a proprietary method, adequate validation is required. Each testing center must be certified by internal quality assurance (IQA) and external quality assurance (EQA) procedures and demonstrate assay validation. QA, validation, proficiency testing, training to standardized techniques, and adequate infrastructure for appropriate sample handling are required*.

### Tissue for MRD assessment

In both PB and BM, MRD status is strongly prognostic for PFS and OS in patients with CLL treated with first-line CIT (Table 1). The multi-compartment nature of CLL (PB, BM, lymph nodes, liver, spleen), however, suggests the possibility of discordant MRD results when sampling different tissues; thus, the sampling site may affect its prognostic ability. Timing of sampling (along the course of disease) is also critical. Concordance between PB and BM MRD status is ~85% at the 10^−4^ threshold and can be affected by treatment type [18–20, 25, 35]. Based on clinical trials, BM assessment may be necessary for U-MRD with certain monoclonal antibody-containing regimens where a relevant discrepancy between PB and BM exists. Studies have shown low PB and BM concordance for patients receiving alemtuzumab (48.6% concordance) [25] or rituximab (79% concordance; 0.7 log lower disease in PB compared to BM) [18, 35]. BM is the most sensitive source for MRD assessment following CIT [25]. Residual disease in spleen, liver, and lymph nodes may, however, play a role in relapse. Current testing methods do not assess these sites [36].

#### Consensus recommendations

*In clinical trials aimed at disease eradication, MRD status should be assessed in both PB and BM. Designing strategies and/or more sensitive techniques allowing PB to be used to monitor MRD is desirable. MRD assessment in PB is useful for screening and informing BM aspiration decisions. If MRD is detected in blood, no BM aspiration is needed. PB U-MRD, however, calls for BM aspiration for confirmatory purposes in regimens where a relevant discrepancy between PB and BM exists. Novel strategies to detect MRD across all disease compartments should be developed, and the utility of circulating cell-free DNA as an MRD measure should be explored. The interchangeability of various MRD methods, tissues, and compartments should be investigated*.

### Timing and frequency of MRD assessment

Most CLL trials reporting MRD data evaluated CIT regimens (Table 1). Targeted therapy MRD data are limited, as are data on optimal timing for MRD testing and MRD kinetics data. Identifying the optimal clinically relevant timepoint for MRD testing requires a greater understanding of CLL kinetics, including MRD clearance and re-emergence and timing of relapse following U-MRD achievement.

#### Consensus recommendations

*For fixed-duration therapies, MRD testing should be aligned with response assessment, at least 2 months after completion of the last treatment* [14]*. For continuous treatment, MRD status should be tested when best clinical response has been achieved; fixed testing timepoints are recommended for clinical trial design. Importantly, patients with PR may achieve U-MRD; therefore, MRD assessment should not be limited to patients with CR. Prospective studies evaluating time-to-U-MRD and time-to-MRD relapse should be conducted to determine their value as secondary endpoints. Measuring clonal growth kinetics by serial MRD assessment could be important in considering U-MRD as a surrogate marker for PFS in fixed-duration therapies. Clinical trials should assess MRD kinetics and its correlation with time-to-event outcomes*.

## MRD in response assessment

### U-MRD significance

**Fig. 1:**
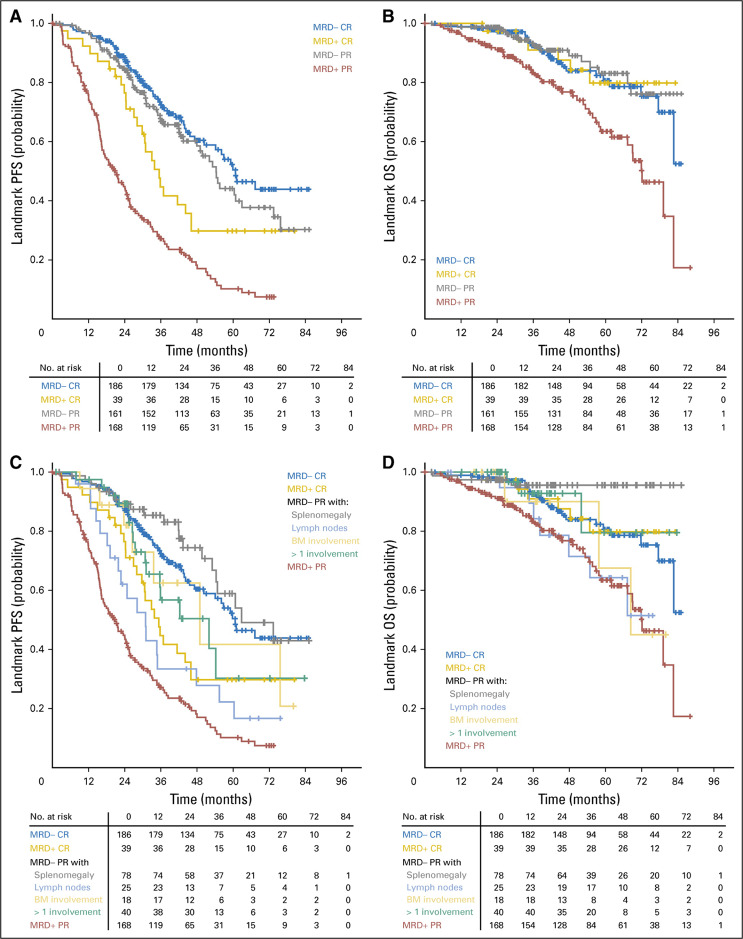
Landmark analysis in the German CLL Study Group CLL8 and CLL10 trials. PFS (**A** and **C**) and OS (**B** and **D**) at end of treatment by PB MRD and additional response status. Reprinted with permission from Kovacs G, Robrecht S, Fink AM, et al. Minimal residual assessment improves prediction of outcome in patients with chronic lymphocytic leukemia (CLL) who achieve partial response: comprehensive analysis of two-phase III studies of the German CLL Study Group. *J Clin Oncol*. 2016;31:3758–3765. BM, bone marrow; CR, complete response; MRD-, minimal residual disease negative; MRD+, minimal residual disease positive; OS, overall survival; PFS, progression-free survival; PR, partial response.

In first-line CIT treatment of CLL, MRD status was independently associated with extended treatment-free survival, PFS, and OS [19, 21–24, 37–39]. U-MRD is more accurately associated with survival than conventional responses after first-line CIT. In this setting, patients achieving U-MRD and PR may have a prognosis superior to that of MRD-positive patients achieving CR (Fig. 1) [21]. The impact of MRD status on treatment outcome after first-line CIT may, however, differ by disease biology (e.g., IGHV mutation status) [24].

In relapsed/refractory CLL, U-MRD status correlates with longer PFS and OS [39–42]. These benefits, however, may be less than those seen in the first-line. Additional trial data are needed regarding MRD status correlation with survival benefit for novel agents and combinations.

#### Consensus recommendations

*The relationship between clinical response (i.e., iwCLL response) and end-of-treatment MRD status requires clarification. Because this may differ between treatment strategies, it should be evaluated in a treatment-specific context. The relationship between end-of-treatment MRD status and PFS and OS may also depend on other factors. Further studies are needed to clarify effects of prior treatment, prior response*, IGHV *mutational status, del(17p)/mutated* TP53 *status, cytogenetic abnormalities, and other variables*.

### U-MRD as a potential surrogate endpoint

In first-line, defined-length CLL treatment, a prognostic association exists between U-MRD and improved outcomes [19, 21, 23, 24, 43, 44]. In relapsed/refractory CLL, preliminary evidence suggests a valid association between U-MRD and improved outcomes with BCL-2 inhibitor treatment (venetoclax ± rituximab), irrespective of del(17p) status [41, 42, 45, 46]. To date, no correlation between MRD status and time-to-event outcomes has been established for B cell receptor (BCR) inhibitor monotherapy or combinations with CD20 mAbs. BCR inhibitor-based treatment is uncommonly associated with U-MRD and is continuous and indefinite, which likely impacts this.

The value of U-MRD as a surrogate for time-to-event endpoints has not been proven in prospective trials. Consequently, MRD is currently not accepted as a surrogate endpoint by regulatory authorities. European Medicines Agency guidelines for anticancer medicinal products state that U-MRD in patients with CLL who are in clinical CR may be used as an intermediate endpoint for licensure in randomized well-controlled studies designed to show PFS superiority provided certain conditions are met [15]. Outside of clinical trials and the setting of allotransplantation, evidence is insufficient to support modification of patient management based on MRD assessment.

#### Consensus recommendations

*Clinical trials should investigate U-MRD rates for different therapies, U-MRD duration, and its impact on outcome. The role of MRD as a surrogate for PFS, specific to treatment type, needs validation, requiring MRD assessment in as many trials as possible, independent of the likelihood of achieving U-MRD. Trials should evaluate whether U-MRD is more reliable in predicting outcomes than clinical response and whether MRD-driven management modifications lead to improved outcomes. U-MRD status may be a suitable endpoint in trials designed to evaluate depth of response (even in the absence of a comparator), and because of its correlation with PFS, it may also be used in trials of fixed-duration treatments to inform treatment-stopping decisions. Patients should be followed until progression and next CLL treatment*.

### Optimal method of response assessment in CLL

Although clinical response and MRD are correlated, each is independently prognostic for outcome [18, 19, 21]. With CIT, MRD is more strongly prognostic than clinical response for PFS [18, 19, 21]. Clinical response has varying significance, depending on MRD status [21].

#### Consensus recommendations

*In clinical practice, the optimal response assessment method depends on individual patient status, type of treatment, and treatment goal. At a minimum, response assessment should include full blood count and clinical examination. BM examination and CT scan may also be included. In clinical trials, BM examination and CT scan should be assessed, and MRD assessment is recommended to inform prognosis and quality of response, and to potentially identify candidates for MRD-driven changes in treatment duration* [23, 37, 47].

### Treatments inducing U-MRD in CLL (Table 4)

Fludarabine, cyclophosphamide, and rituximab (FCR) treatment has a high likelihood of inducing U-MRD [19, 48–50]. First-line treatments inducing U-MRD include chlorambucil and obinutuzumab [44], chlorambucil and ofatumumab [51], bendamustine and rituximab [48], bendamustine and obinutuzumab [52, 53], and fludarabine, cyclophosphamide, and obinutuzumab [54]. BCR pathway inhibitor-based therapy (ibrutinib, idelalisib) rarely results in U-MRD status [55], but early data indicate that ibrutinib and venetoclax ± obinutuzumab can lead to U-MRD [9, 12, 56]. Venetoclax and CD20 mAbs can lead to U-MRD in first-line and relapsed/refractory CLL [57–61].

**Table 4:** Representative treatments shown to induce MRD in CLL.

Study	Line	Agent	Type	*N*	Assay	U-MRD
Goede et al. [44]	First	G + clb vs R + clb	Ph 3	474	ASO-PCR	GClb:19.5% (BM), 37.7% (PB) RClb: 2.6% (BM), 3.3% (PB)
Hillmen et al. [51]	First	Ofa + clb vs Clb	Ph 3	212	NS	Ofa + clb: 8% (BM or PB)
Eichhorst et al. [48]	First	BR vs FCR	Ph 3	561	Flow MRD4	BR: 11% (BM) FCR: 27% (BM)
Sharman et al. [52]	First	BG	Ph 2	102	Flow MRD4	Best response: 75.5% (PB)
Stilgenbauer et al. [53]	First	BG	Ph 3b	158	Flow MRD4	27.8% (BM), 59.5% (PB)
Leblond et al. [54]	First	GFC	Ph 3	140	Flow MRD4	35.7% (BM), 64.3% (PB)
Böttcher et al. [19]	First	FCR vs CF	Ph 3	493	Flow MRD4	FCR: 63% (PB) CF: 35% (PB)
Eichhorst et al. [48]	First	BR vs FCR	Ph 3	564	Flow MRD4	BR: 11% (BM), 38% (PB) FCR: 27% (BM), 49% (PB)
Dartigeas et al. [80]	First	FCR ± R maintenance	Ph 3	542	Flow MRD5	36.7% (BM), 59.3% (PB)
Munir et al. [50]	First	FCR vs FCRM	Ph 2b	215	Flow MRD4	FCR: 50.5% (BM) FCRM: 43.5% (BM)
Wierda et al. [9]	First	Ibr + ven	Ph 2	163	Flow MRD4	82% (PB)
Rogers et al. [56]	First	G + ibr + ven	Ph 2	25	Flow MRD4	60% (BM), 72% (PB)
Fischer et al. [71]	First	G + ven vs G + clb	Ph 3	432	ASO-PCR	G + ven: 75.5% (PB), 56.9% (BM) G + clb: 35.2% (PB), 17.1% (BM)
Stilgenbauer et al. [58]	Any	Ven + BR or BG	Ph 1b	17 (BR) 8 (BG)	Flow MRD4	Ven + BR: 67% (NS) Ven + BG: 50% (NS)
Cramer et al. [59]	Any	B, then ven + G	Ph 2	63	Flow MRD4	1 L: 12% (BM), 91% (PB) R/R: 14% (BM), 83% (PB)
Burger et al. [63]	Any	Ibr vs Ibr + R	Ph 2	208	Flow MRD4	Ibr: 12 mo, 34.4% (BM), 24 mo, 19.8% (BM); Ibr + R: 12 mo, 18.5% (BM) 24 mo, 12.2% (BM)
Stilgenbauer et al. [42]	Any	Ven	Ph 2	158	Flow MRD	12.7% (BM), 30% (PB)
Rawstron et al. [8]	R/R	Ibr + G	Ph 1	40	MRD4	Ibr-naïve: 30% (PB) Prior ibr: 60% (PB)
Roberts et al. [64]	R/R	Ven	Ph 1	116	MRD4	5% (BM)
Fraser et al. [7]	R/R	Ibr + BR vs PBO + BR	Ph 3	578	Flow MRD4	Ibr + BR: 26.3% (PB or BM) PBO + BR: 6.2% (PB or BM) *P* < .0001
Seymour et al. [41]	R/R	Ven + R vs BR	Ph 3	389	Flow MRD4 ASO-PCR	VenR: 27.3% (BM), 83.5% (PB) BR: 1.5% (BM), 23.1% (PB)

*1L* first-line, *ASO-PCR* allele-specific oligonucleotide polymerase chain reaction, *BG* bendamustine and obinutuzumab, *BM* bone marrow, *BR* bendamustine and rituximab, *CF* fludarabine and cyclophosphamide, *Clb* chlorambucil, *Flow* flow cytometry, *FCR* fludarabine, cyclophosphamide, and rituximab, *FCRM* FCR and mitoxantrone, *G* obinutuzumab, *GFC* obinutuzumab, fludarabine, and cyclophosphamide, *Ibr* ibrutinib, *MRD* measurable residual disease, *PB* peripheral blood, *Ofa* ofatumumab, *PBO* placebo, *Ph* phase, *R* rituximab, *Ven* venetoclax.

In relapsed/refractory CLL, CIT can sometimes induce U-MRD [62]. Ibrutinib combined with rituximab or obinutuzumab, or with bendamustine and rituximab, can induce U-MRD [7, 8, 63]. Venetoclax monotherapy can achieve U-MRD in patients with or without del(17p) [42, 64], but higher rates have been reported for venetoclax combined with either a CD20 mAb or ibrutinib [41, 61]. Data on U-MRD durability for most therapies are lacking.

#### Consensus recommendations

*Clinical trials of defined-duration CLL treatments should assess MRD at least 2 months after the last treatment cycle is completed for correlation with PFS and OS*.

### Disease-related factors prognostic of U-MRD

Type of therapy and line of treatment have the strongest associations with achieving U-MRD [22]. CIT, mutated IGHV, wildtype *TP53*, and absence of del(17p) are associated with a higher likelihood of achieving U-MRD [19, 22–24, 49, 65]. Other factors may include cytogenetic abnormalities and age [22, 23, 65].

#### Consensus recommendations

*Factors associated with achieving U-MRD should be identified for each agent/regimen. Collaborative efforts are important for elucidating MRD biology for emerging therapies and correlations with outcomes. Ideally, for each treatment strategy, U-MRD objectives and methods should be defined at treatment initiation*.

### MRD relapse

MRD relapse has not been defined. Reports have used newly detectable disease above the 10^−4^ threshold on two consecutive PB tests [18, 66]. Relapse dynamics may vary according to disease characteristics, prognostic factors, and/or treatment [67]. MRD relapse may be used as a marker of subclinical progression in clinical trials to inform on disease kinetics, treatment re-initiation, and relationship with clinical relapse. In clinical practice, MRD relapse status is not currently used to inform treatment decisions. The potential benefit of early treatment for MRD relapse (versus waiting for clinical relapse) has not been systematically investigated.

#### Consensus recommendations

*Serial MRD testing is not indicated in routine practice; MRD relapse currently has no impact on treatment decisions for standard of care. We propose that it be defined as detectable MRD (*>*10*^*−4*^*) on at least two consecutive timepoints in PB. The optimal time between the two positive tests should be evaluated in trials. Further studies are needed to define what constitutes MRD relapse and should include correlation with subsequent clinical relapse and its timing. Trials evaluating the possible benefit of treating MRD relapse or asymptomatic, progressive disease are needed. These will likely require randomization to therapy versus observation until clinical relapse*.

## Clinical utility of MRD assessment

### MRD in clinical practice versus clinical trials

Current guidelines do not recommend routine MRD testing in clinical practice [67, 68]. One trial demonstrated that patients achieving U-MRD early in first-line CIT treatment have a prognosis similar to those achieving U-MRD later in treatment [23]. Two trials demonstrated that patients achieving U-MRD had a longer duration of PFS compared to patients with detectable MRD, suggesting that MRD may be a prognostic factor with some therapies [60, 61]. Lacking further prospective studies, however, this is insufficient evidence to change clinical practice. Data relating MRD and outcomes for novel nonchemotherapeutic treatments are limited.

#### Consensus recommendations

*In clinical trials, MRD may be explored for modifying treatment duration or for determining whether switching treatment strategies may be beneficial. To transition MRD assessment from trials into routine clinical practice, however, data demonstrating that such modifications lead to improved outcomes are needed*.

### U-MRD and quality of life (QoL)

Currently, no data associate U-MRD with QoL. Potentially, using U-MRD to inform when to stop long-term therapy could impact patient QoL. U-MRD is a prerequisite for curing CLL but does not in itself indicate cure.

#### Consensus recommendations

*Data from CLL studies that included MRD and QoL assessments should be retrospectively analyzed for a possible link. Future trials assessing MRD status should evaluate the effect of achieving U-MRD on QoL parameters and include CLL-adapted QoL questionnaires. Durable disease control with minimal toxicity and improved QoL without U-MRD achievement may be a suitable treatment goal but requires clinical validation*.

### Future MRD utility

MRD status offers a highly sensitive endpoint that may be used to design novel treatment strategies.

#### Consensus recommendations

*The usefulness of MRD status in clinical practice depends on available therapies and setting as well as the cost and infrastructure required for assessing MRD. Currently, there is no clear role for MRD assessment in routine practice. Once sufficient data are available, MRD status may potentially inform decisions on when to stop or adjust treatment. Trial data for MRD and “omics” may prove useful in elucidating the most effective treatments and differentiating between subgroups. Clinical trials with fixed-duration treatment should be designed to achieve the deepest remission in the highest proportion of patients, evaluating MRD status with a test in PB at a sensitivity of at least 10*^*−4*^ *to correlate with time-to-event endpoints*.

## Discussion

Advances in bioanalytics have provided sensitive techniques for quantifying depth of response in patients with CLL. A large body of evidence indicates that MRD status is independently prognostic for time-to-event outcomes (Table 1). Its significance in CLL is complex and multifactorial, but MRD testing has already become widespread in CLL clinical trials. Future studies will determine whether MRD status may inform treatment decisions and/or represents an accepted surrogate endpoint in clinical trials of new CLL therapies. The current utility and future potential of MRD in CLL underscore the need to use standardized, validated assays; adopt a routine, unambiguous nomenclature; and report assay results systematically. This international panel was convened to provide expert guidance on these issues.

We strongly recommended that trials of fixed-duration treatments designed to achieve deepest remission in the highest proportion of patients evaluate MRD status in PB at a sensitivity of at least 10^−4^ to correlate with time-to-event outcomes. At present, however, there is no clear role for MRD status determination in routine CLL clinical practice.

CLL treatment has markedly evolved over the last 10 years. Alkylating-agent and purine analog monotherapies have developed into combination chemotherapy. Subsequently, CD20 mAbs have been added. The most active CIT regimen, FCR, achieved CR in the majority of patients, with U-MRD in approximately one-half of first-line patients [48]. A challenge with FCR, however, is myelosuppression, limiting its use to younger, fit patients, and long-term toxicities can be concerning. Approximately 50–55% of treated first-line patients with mutated-IGHV are progression-free for >10 years post-therapy and may be cured [24]. These outcomes initiated the focus on MRD as an important endpoint.

Focus, however, shifted with development of the BCR signaling inhibitors ibrutinib and idelalisib [6, 69, 70]. These achieved extremely durable disease control, including in high-risk, relapsed/refractory patients, and even more durable responses in the first-line. Most patients achieved remission, but most responses were partial, and treatment needed to be administered indefinitely. Interest in U-MRD as an endpoint consequently waned.

More recently, venetoclax, a BCL2 inhibitor that potently induces apoptosis in CLL cells, was developed [57, 61, 64]. It is highly effective in eliminating disease and is FDA-approved for treatment of adults with CLL or small lymphocytic leukemia. Venetoclax may be used for continuous monotherapy or for fixed-duration treatment when combined with a CD20 mAb. Venetoclax-based treatment not only achieves a higher rate of U-MRD in first-line and relapsed CLL than CIT does, but it also is well-tolerated, including in older patients, making deep remission and U-MRD with fixed-duration treatment a realistic goal [41, 71]. Thus, the interest and clinical importance of MRD as a treatment objective are more important than ever for patients and clinicians.

## Supplementary information

Supplemental Material
