# Supplementary material for: Measurable residual disease in chronic lymphocytic leukemia: expert review and consensus recommendations
Source: Leukemia. 2021 Jun 24;35(11):3059–72. doi: 10.1038/s41375-021-01241-1 (PMC8550962; doi:10.1038/s41375-021-01241-1)
Supplement: Supplementary file 1 — Supplemental Material [file 41375_2021_1241_MOESM1_ESM.pdf]

SUPPLEMENTARY APPENDIX

International Steering Committee Members

Dr. William Wierda, United States; Prof. Xavier Badoux, Australia; Dr. Jennifer Brown, United States; Prof. Florence Cymbalista, France; Prof. Paolo Ghia, Italy; Prof. Peter Hillmen, United Kingdom; Dr. Andrew Rawstron, United Kingdom; and Prof. Stephan Stilgenbauer, Germany

Members of National Faculties

Dr. Virginia Abello, Colombia; Dr. Ahmed Al-Absi, Saudi Arabia; Dr. Hani Al-Hashmi, Saudi Arabia; Dr. Arif Allam, United Arab Emirates; Dr. Celso Arrais, Brazil; Prof. Onder Arslan, Turkey; Dr. Carlos Avendaño, Guatemala; Prof. Osnat Bairey, Israel; Dr. Versha Banerji, Canada; Prof. Mario Bargetzi, Switzerland; Dr. Fernando Barroso Duarte, Brazil; Dr. Ali Bazarbachi, Lebanon; Dr. Fernando Bezares, Argentina; Dr. Adrian Bloor, United Kingdom; Prof. Csaba Bödör, Hungary; Dr. Sebastian Boettcher, Germany; Dr. Francesc Bosch, Spain; Prof. André Bosly, Belgium; Dra. Carmen Cao, Chile; Dr. José Carda, Portugal; Dr. Eduardo Cervera, Mexico; Dr. Cheng-Shyong Chang, Taiwan; Dr. Maria de Lourdes Chaufaille, Brazil; Dr. Carlos Chiattone, Brazil; Dr. Naeem Choudari, Saudi Arabia; Dr. Poulsen Christian Bjørn, Denmark; Dr. Ilse Christiansen, Denmark; Dr. Guillermo Conte, Chile; Prof. Paolo Corradini, Italy; Dr. Rita Coutinho, Portugal; Dr. Gavin Cull, Australia; Dr. Tamayra Cumba, Dominican Republic; Prof. Antonio Cuneo, Italy; Dr. Rosario Custidiano, Argentina; Dr. Javier De La Serna, Spain, Prof. Fatih Demirkan, Turkey; Dr. Ricardo De Sá Bigni, Brazil; Dr. Sigrid De Wilde, Belgium; Prof. Michael Doubek, Czech Republic; Dr. Peter Dreger, Germany; Dr. Alexander Egle, Austria; Prof. Ki-Seong Eom, Korea; Dr. German Espino, Panama; Dr. Ronan Foley, Canada; Dr. George Follows, United Kingdom; Dr. Chris Fox, United Kingdom; Dr. Raúl Gabús, Uruguay; Prof. Gianluca Gaidano, Italy; Dr. Kenny Mauricio Galvez, Colombia; Prof. Paolo Ghia, Italy; Prof. Krzysztof Giannopoulos, Poland; Prof. Maria Gomes da Silva, Portugal; Dr. David Gómez-Almaguer, Mexico; Prof. David Gottlieb, Australia; Dr. Nelson Hamerschlak, Brazil; Dr. Amjad Hayat, Ireland; Dr. Dominik Heim, Switzerland; Prof. Manfred Hensel, Germany; Dr. Yair Herishanu, Israel; Dr. Jose Angel Hernández, Spain; Prof. Ulrich Jäger, Austria; Prof. Ann Janssens, Belgium; Dr. Nathalie Johnson, Canada; Dr. Takizawa Jun, Japan; Prof. Suzumiya Junji, Japan; Dr. Veli Kairisto, Finland; Prof. Arnon P. Kater, Netherlands; Dr. Bonnie Kho, Hong Kong; Prof. Ong Kiat Hoe, Singapore; Prof. Jin Seok Kim, Korea; Prof. Eva Kimby, Sweden; Dr. Michael Knauf, Germany; Dr. Jorgen Kristensen, United Arab Emirates; Prof. Bryone Kuss, Australia; Dr. Anton W. Langerak, Netherlands; Dr. Sarah Lawless, United Kingdom; Dr. Rock Leung, Hong Kong; Dr. Vesa Lindström, Finland; Dr. Herman Liu, Hong Kong; Dr. Diego Emilio Lopera Cortez, Colombia; Dr. Marco Lopez, Peru; Dr. Javier Loscertales, Spain; Dr. Edmond Ma, Hong Kong; Dr. David MacDonald, Canada; Dr. Marie Maerevoet, Belgium; Dr. Vivian Mak, Hong Kong; Prof. Paula Marlton, Australia; Dr. Helen Marr, United Kingdom; Dr. Mattias Mattsson, Sweden; Prof. Francesca Mauro, Italy; Dr. Alison McCaig, United Kingdom; Dr. Helen McCarthy, United Kingdom; Dr. Luis Meillon, Mexico; Dr. Gábor Mikala, Hungary; Prof. Stefano Molica, Italy; Prof. Stephen Mulligan, Australia; Dr. Pablo Muxi, Uruguay; Dr. Juan Ramon Navarro Cabrero, Peru; Prof. Peter Neumeister, Austria; Dr. Carsten Niemann, Denmark; Prof.

Eugene Nikitin, Russia; Dr. Mauricio Ocqueteau, Chile; Prof. Stephen Opat, Australia; Dr. Derville O'Shea, Ireland; Dr. Roberto Ovilla, Mexico; Dr. Carolyn Owen, Canada; Prof. Panayiotis Panayiotidis, Greece; Dr. Ramesh Pandita, Kuwait; Prof. Eleni Papadaki, Greece; Dr. Piers Pattern, United Kingdom; Dr. Miguel Pavlovsky, Argentina; Dr. Colin Phipps Diong, Singapore; Prof. Regina Pileckyte, Lithuania; Dr. Belinda Pinto Simões, Brazil; Prof. Sarka Pospisilova, Czech Republic; Dr. Jose Ramón Rivas Llamas, Mexico; Prof. Christoph Renner, Switzerland; Dr. Suzuki Ritsuro, Japan; Prof. Tadeusz Robak, Poland; Dr. Myriam Rodriguez, Colombia; Dr. Davide Rossi, Switzerland; Prof. Aoki Sadao, Japan; Dr. Tommi Salmi, Finland; Dr. Cesar Augusto Samanez Figari, Peru; Dr. Julio Sanchez Ávalos, Argentina; Prof. Lev Shvidel, Israel; Prof. Teoman Soysal, Turkey; Dr. Martin Spacek, Czech Republic; Dr. Kostas Stamatopoulos, Greece; Prof. Michael Steurer, Austria; Prof. Stephan Stilgenbauer, Germany; Dr. Yu-Chieh Su, Taiwan; Prof. Constantine Tam, Australia; Dr. Daryl Tan, Singapore; Dr. María José Terol, Spain; Dr. Hoa Thi Tuyet Tran, Norway; Prof. Geir Tjønnfjord, Norway; Dr. Patrick Thornton, Ireland; Dr. Cyndy Toze, Canada; Prof. Eric Tse, Hong Kong; Prof. Levent Undar, Turkey; Dr. Maria Soledad Undurraga, Chile; Prof. Elisabeth Vandenberghe, Ireland; Dr. Vanessa Van Hende, Belgium; Dr. Jorge Vaz Pinto Neto, Brazil; Dr. Jorge Vela Ojeda, Mexico; Dr. Alberto Villalobos, Mexico; Prof. Michael-Clemens Wendtner, Germany; Prof. Thomas Wolff, Germany; Dr. Shang-Ju Wu, Taiwan; Dr. Sze-Fai Yip, Hong Kong; Prof. Andrey Zaritskey, Russia.

Supplementary Figure 1. Consensus Report Development Process

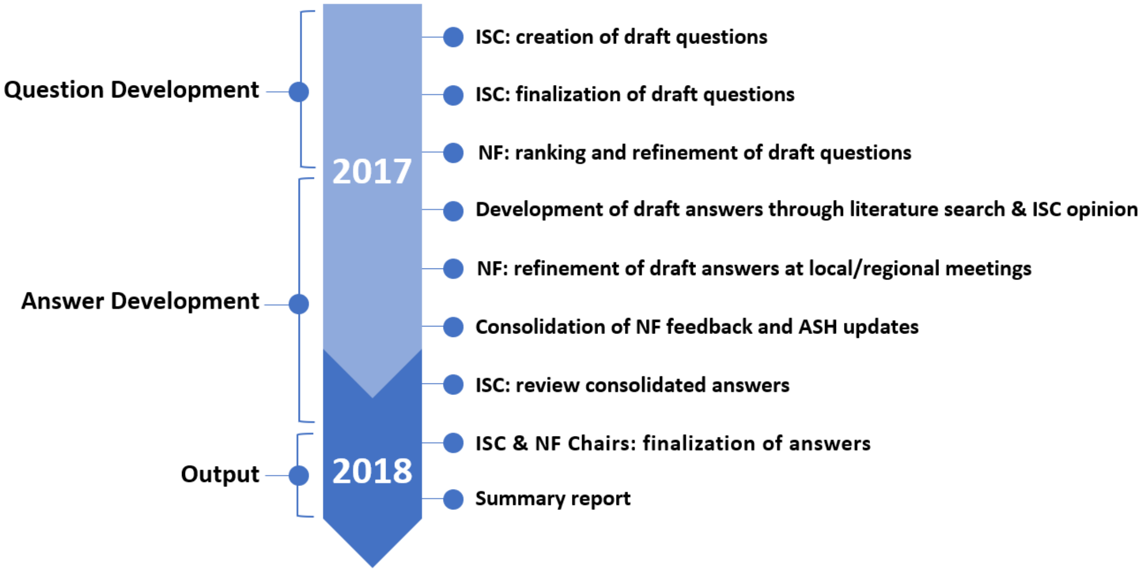

ASH, American Society of Hematology; ISC, international steering committee; NF, National Faculty
